# Antimalarial chloroquine metamorphosed into antiviral agent against HIV with four modes of actions

**DOI:** 10.1186/1471-2334-12-S1-O21

**Published:** 2012-05-04

**Authors:** M Chandramohan, P Selvam, SC Vivekananthan, D Sivakumar, E De Clercq

**Affiliations:** 1 Kamarajar Jaundice, Liver Hospital & Research Centre, Madurai-625001, Tamil Nadu, India; 2 Devaki Amma Memorial College of Pharmacy, Chelembra, Malapuram-673436, Kerala, India; 3 Madurai Medical College, Madurai-625020, India; 4 Rega Institute of Medical Research, Katholieke University, B-3000, Leuvan, Belgium

## Background

To identify an economical and effective antiviral agent we are nurturing chloroquine (CQ) which is an antiviral agent with multimodal and immune modalatory actions. Earlier studies have reported inhibition of HIV by CQ through its anti-integrase activity and also had elucidated that CQ inhibits HIV by inhibiting glycosylation of envelope glycoprotein. It had also been shown that CQ blocked Tumor-Necrosis-Factor (TNF-) alpha and Interleukin-6(IL-6); production and interferes with HIV replication.

## Method

In vitro assay of anti HIV activity of CQ in MT4-cells and clinical study.

## Results

We observed Inhibitory Concentration (IC50) 7.19ug/ml and Cytotoxic-Concentration (CC50) >35.43 and the maximum protection noted was 90; a laudable observation. Results on hand; we launched a preliminary study of giving CQ at 2 tables per day as add-on drug along with other antiretroviral agents to 6 HIV patients proved by ELISA and Western blot; age ranged between 6 and 48. We have observed CD4 and CD8 cells count improved by 20% to 35% but their sustaining effects couldn’t be assessed because two patients died due to overwhelming intercurrent infection and four patients had good improvement for 2-3 yr and lost for further follow up. This report of ours is next to first Indian report.

## Conclusion

We could ascertain that this add-on drug CQ had been tolerated well and major side effects were not observed and with good anti HIV action.

